# Links between copper and cholesterol in Alzheimer's disease

**DOI:** 10.3389/fphys.2013.00111

**Published:** 2013-05-16

**Authors:** Ya Hui Hung, Ashley I. Bush, Sharon La Fontaine

**Affiliations:** ^1^Oxidation Biology Laboratory, Florey Institute of Neuroscience and Mental HealthParkville, VIC, Australia; ^2^Centre for Neuroscience Research, The University of MelbourneParkville, VIC, Australia; ^3^Department of Pathology, The University of MelbourneParkville, VIC, Australia; ^4^Strategic Research Centre for Molecular and Medical Research, Deakin UniversityBurwood, VIC, Australia; ^5^Centre for Cellular and Molecular Biology, School of Life and Environmental Sciences, Deakin UniversityBurwood, VIC, Australia

**Keywords:** copper, cholesterol, Alzheimer's disease, ApoE, amyloid precursor protein, Aβ, tau, lipid rafts

## Abstract

Altered copper homeostasis and hypercholesterolemia have been identified independently as risk factors for Alzheimer's disease (AD). Abnormal copper and cholesterol metabolism are implicated in the genesis of amyloid plaques and neurofibrillary tangles (NFT), which are two key pathological signatures of AD. Amyloidogenic processing of a sub-population of amyloid precursor protein (APP) that produces Aβ occurs in cholesterol-rich lipid rafts in copper deficient AD brains. Co-localization of Aβ and a paradoxical high concentration of copper in lipid rafts fosters the formation of neurotoxic Aβ:copper complexes. These complexes can catalytically oxidize cholesterol to generate H_2_O_2_, oxysterols and other lipid peroxidation products that accumulate in brains of AD cases and transgenic mouse models. Tau, the core protein component of NFTs, is sensitive to interactions with copper and cholesterol, which trigger a cascade of hyperphosphorylation and aggregation preceding the generation of NFTs. Here we present an overview of copper and cholesterol metabolism in the brain, and how their integrated failure contributes to development of AD.

## Introduction

Since the first case description of Alzheimer's disease (AD) by Alois Alzheimer, AD has steadily gained increasing recognition as the most common neurodegenerative disorder worldwide, and as such, it has been the focus of intense biomedical research efforts. The main brain regions affected in AD include the enterohinal cortex, hippocampus, basal forebrain and amygdala, which exhibit synaptic loss resulting in extensive brain atrophy (Mattson, 2004). Currently, confirmation of an AD diagnosis requires post-mortem examination of the brain for the presence of extracellular amyloid plaques and intracellular neurofibrillary tangles (NFTs) (Alzheimer, 1907), which are the key identifying pathological signatures of AD. Clinically, AD patients present with symptoms of memory loss, altered personality and behavior, and impaired executive function. Medical advances that have contributed to increased longevity and an aging world population is propelling the number of AD cases on a trajectory to quadruple to 106.2 million over the next 40 years (Brookmeyer et al., 2007). This is projected to lead to an ~500% increase in medical and health care costs (http://www.alz.org). In addition, AD can cost patients' family and friends their physical and emotional health, as well as significant financial loss. Therefore, there is a huge demand for an effective disease-modifying therapy for AD that will extend the productive and healthy lifetime of individuals, and reduce its negative socio-economic impact on society.

AD cases can be broadly classified as familial or sporadic. Early-onset familial AD (FAD), which comprises 5–10% of AD cases, is caused by genetic mutations in the genes encoding the amyloid precursor protein (*APP*) and presenilins 1 and 2 (*PSEN1* and *PSEN2*). The remaining AD cases are late-onset and sporadic, with diverse biochemical and clinical presentations. Common to both types of AD is the increased level of Aβ peptide, especially the neurotoxic Aβ_42_ species, as a consequence of enhanced cleavage of APP by β- and γ-secretases. The complex and multifactorial biochemistry that underlies sporadic AD presents a challenge to the discovery of a specific etiology and hence the development of an effective universally applicable therapy. Aging is acknowledged as the leading risk factor for AD, with numerous genetic and environmental factors also found to associate with AD (Pappolla et al., 2003; Cannon and Greenamyre, 2011; Hollingworth et al., 2011; Naj et al., 2011; Bertram and Tanzi, 2012). However, detailed understanding of the contribution of most of these risk factors to the etiology of sporadic AD is yet to be achieved. Amongst the various risk factors for AD, corrupted copper and cholesterol homeostasis have attracted considerable attention independently over the last two decades as potential therapeutic targets. Both copper and cholesterol are critical to normal brain development and function, which include neurotransmission, myelination, and synaptogenesis. Mounting evidence indicates a connection between copper and cholesterol pathways in the pathogenesis of AD. Here we present an overview of copper and cholesterol metabolism in the brain, and the first description of how their integrated failure may lead to the development of AD.

## Brain copper homeostasis

There is an increasing appreciation of a role for copper in normal brain development and function. The importance of copper for normal brain function is underscored by the profound neurodegeneration in Menkes and Wilson diseases, rare genetic disorders of copper deficiency and overload, respectively (Burkhead et al., 2011; Kaler, 2011). More commonly, copper dyshomeostasis is evident in a number of major neurodegenerative disorders, which include AD and Parkinson's disease (PD). Compromised copper regulation in these disorders potentiates inappropriate interactions with aggregation-prone proteins such as Aβ and α-synuclein, respectively, to form neurotoxic copper-protein aggregates (Atwood et al., 1998, 2004; Drew et al., 2008; Gunn et al., 2012; Camponeschi et al., 2013).

The estimated copper content in a healthy human adult brain is 7–10% of total body copper, similar to that found in the liver, the major organ of copper homeostasis (Cartwright and Wintrobe, 1964; Linder, 1991). The redox cycling of copper between Cu^2+^ and Cu^+^ oxidation states is exploited by many metabolic processes to catalyze various enzyme reactions. Some of the key cuproenzymes include ceruloplasmin (CP; iron transport and radical scavenging), Cu,Zn-superoxide dismutase 1 (SOD1; antioxidant defense), dopamine-β-hydroxylase (neurotransmission), cytochrome *c* oxidase (CCO; electron transport and oxidative phosphorylation), and tyrosinase (pigmentation) [reviewed in (Linder and Hazegh-Azam, 1996; Nevitt et al., 2012)]. Paradoxically, the utilization of copper's redox activity in Fenton or Haber–Weiss reactions promotes the production of toxic reactive oxygen species (ROS) (Halliwell and Gutteridge, 1984). Oxidative stress, such as that evident in AD, ensues when the production of ROS overwhelms available antioxidant defenses. The brain is particularly vulnerable to oxidative stress with disproportionately low levels of antioxidants relative to its high rate of oxidative metabolism (Floyd, 1999). Therefore, the dichotomous nature of copper demands a precise regulation to maintain an appropriate level and distribution in the brain, and to prevent inadvertent interactions with other cellular components.

In a human adult brain, copper is particularly enriched in the hippocampus (Dobrowolska et al., 2008) and substantia nigra (Davies et al., 2013). In the hippocampus, following neuronal depolarization, about 15 μM of copper is released into the glutamatergic synaptic cleft from synaptic vesicles (Rajan et al., 1976; Hartter and Barnea, 1988; Kardos et al., 1989; Barnea et al., 1990; Hopt et al., 2003). This release of copper during neurotransmission involves copper-independent trafficking of the copper transporter ATP7A to neuronal processes after glutamate excitation of synaptic *N*-methyl-D-aspartate (NMDA) receptors (Schlief et al., 2005, 2006). This ATP7A-mediated copper release has been proposed to be a neuroprotective mechanism, whereby synaptic copper catalyzes S-nitrosylation of NMDA receptors to down-regulate their activity, and thus protects neurons from excitotoxicity (Schlief et al., 2006). The ability of copper to mediate suppression of long-term potentiation (LTP) in hippocampal slices further supports a role for copper as a neuromodulator (Doreulee et al., 1997). In contrast to a role for copper in hippocampal glutamatergic synapse neurotransmission, the role of copper in the substantia nigra remains obscure. In the substantia nigra, there is a concomitant elevation of copper, iron and zinc compared to other brain regions (Davies et al., 2013). The substantia nigra is therefore, more susceptible to oxidative stress that can be induced by redox-active copper and iron. Paradoxically, greater concentration of copper and zinc is likely to be associated with a higher demand for copper- and zinc-dependent antioxidant activity of SOD1 in the substantia nigra to counter oxidative stress (Saggu et al., 1989; Davies et al., 2013).

### Brain copper import and export

Copper is generally acquired through dietary intake of copper-rich foods such as animal liver, nuts, and shellfish. Dietary copper (Cu^2+^) is absorbed into the body by intestinal mucosal cells, and delivered to the liver via the portal blood bound to albumin, transcuprein, amino acids, and small peptides (Bearn and Kunkel, 1954; Neumann and Sass-Kortsak, 1967; Weiss and Linder, 1985; Wirth and Linder, 1985). In hepatic cells, copper is incorporated into CP, which carries the majority of copper in the blood for circulation to extrahepatic tissues (Campbell et al., 1981; Lee et al., 1993). Excess dietary copper is secreted into the bile from hepatic cells via biliary canaliculi and eliminated in feces.

Emerging studies of brain copper homeostasis is beginning to shed light on the mechanism of brain copper import, distribution and export. A concentration difference of up to 100-fold between plasma (11–25 μM) (Tietz, 1987) and cerebrospinal fluid (CSF; ~0.25 μM) copper signifies restricted copper uptake into the brain (Kjellin, 1963; Lentner, 1981). A rat brain perfusion study suggested that non-protein bound free copper ion is the predominant copper species that enters the brain via both the blood-brain barrier (BBB) and the blood-cerebrospinal fluid barrier (BCB) (Choi and Zheng, 2009). The greater uptake of copper into the brain parenchyma compared to CSF suggests that the BBB is the main site through which copper enters the brain. An enrichment of copper transporters CTR1, ATP7A, and ATP7B in the brain capillaries and choroid plexus, implicates their involvement in controlling copper influx into the brain parenchyma and CSF (Iwase et al., 1996; Qian et al., 1998; Kuo et al., 2006; Niciu et al., 2006; Choi and Zheng, 2009; Donsante et al., 2010; Davies et al., 2013).

The high affinity copper transporter CTR1 is the primary gatekeeper of copper entry into the brain via brain capillary endothelial cells that form the BBB and choroid plexus epithelial cells that form the BCB. Consistent with a role for CTR1 in cellular copper uptake, it is localized predominantly at the apical membrane in human choroid plexus epithelial cells (Davies et al., 2013). Furthermore, CTR1 heterozygous mice have lower brain copper concentration relative to wild-type controls, and copper deficiency induces an up-regulation of choroid plexus CTR1 expression in mice (Kuo et al., 2001, 2006; Lee et al., 2001; Gybina and Prohaska, 2006). An alternate route of copper uptake into the brain is via the divalent metal transporter, DMT1 (Arredondo et al., 2003). However, the extent to which DMT1 participates in brain copper import is unclear, particularly in the wake of a recent study disputing copper transport by DMT1 (Illing et al., 2012). In conjunction with CTR1, ATP7A, and ATP7B regulate the rate-limiting step of copper influx into the brain. Immunohistochemical stains revealed that in choroid plexus epithelial cells, ATP7A predominantly localizes to the basolateral membrane, whereas ATP7B localizes to the apical membrane (Davies et al., 2013). The distinct membrane localization of these two homologous copper transporters may be a regulatory mechanism related to their respective enzyme kinetics to provide a strict control over copper transport across the BCB and BBB (Tsivkovskii et al., 2002; Barnes et al., 2005; Hung et al., 2007). The comparatively slower rate of copper movement into the CSF relative to copper influx into choroid plexus may be explained by the presence of kinetically slower ATP7B at the CSF-facing apical membrane of choroid plexus epithelial cells (Choi and Zheng, 2009). The kinetically faster ATP7A is more favorably localized to the basolateral membrane to facilitate copper efflux into the blood (Davies et al., 2013). Therefore, BCB serves as the main exit route for excess copper from the brain. Conversely, BBB as the main entry point for copper into the brain has lower ATP7A expression with a brain capillary-facing basolateral localization to enable rapid but limited import of copper into the brain to counterbalance any sudden brain copper depletion (Choi and Zheng, 2009).

### Intracellular copper transport in brain cells

There have been limited studies on brain cellular copper transport. It has been largely assumed that the homeostatic mechanisms that regulate cellular copper movement in brain cells parallel that of well-defined pathways in peripheral cells. Indeed, recent studies in astrocytes support this notion (Scheiber et al., 2010, 2012). CTR1, in addition to mediating the initial copper uptake into the brain, is implicated also in general copper uptake into neuronal and glial cells. However, differential expression of CTR1 in different brain cells pertains to cell-specific copper requirements (Davies et al., 2013).

To prevent the generation of ROS by free copper ions inside cells, three well-characterized metallochaperone pathways have evolved to escort intracellular copper: (1) ATOX1; (2) CCS; and (3) COX17. The copper chaperone ATOX1 is widely expressed in the brain, which include the hippocampus, thalamus, cerebellum, and corpus callosum (Klomp et al., 1997; Naeve et al., 1999; Davies et al., 2013). It delivers copper to cuproenzymes in the secretory pathway, and in particular, to the copper transporters ATP7A and ATP7B at the *trans*-Golgi network (TGN). In addition to its role as a copper chaperone, it regulates the catalytic activity of ATP7B (Walker et al., 2002). ATOX1 is essential to ATP7A copper efflux activity. It stimulates ATP7A translocation from the TGN to the cell surface via direct interactions and copper exchange (Hamza et al., 2003). Consistent with the original identification of ATOX1 in the yeast *Saccaromyces cerevisae* as an antioxidant protein (Lin and Culotta, 1995), its over-expression in neuronal cell lines confers protection against oxidative insults (Kelner et al., 2000).

The copper chaperone for superoxide dismutase (CCS) loads copper into SOD1 located in the cytoplasm (Culotta et al., 1997). It is expressed in the mammalian brain, but its expression relative to brain regional distribution is unknown (Gybina and Prohaska, 2006). Interestingly, the expression of CCS is sensitive to copper deficiency in the cerebellum, but not in the choroid plexus (Gybina and Prohaska, 2006). In addition to its interactions with SOD1, CCS also transports copper to BACE1 (Angeletti et al., 2005), the β-secretase involved in APP cleavage that results in the production of amyloid plaque-forming Aβ peptide. CCS deficiency promotes Aβ production, which implicates CCS in the regulation of BACE1 activity (Gray et al., 2010). Furthermore, BACE1 competes with SOD1 for binding to CCS, which may contribute to oxidative stress evident in AD (Angeletti et al., 2005; Dingwall, 2007).

COX 17 is the copper chaperone responsible for delivery of copper to COX11, SCO1, and SCO2 in the mitochondria for the metallation of CCO (Glerum et al., 1996a,b; Amaravadi et al., 1997; Beers et al., 1997; Horvath et al., 2000; Horng et al., 2004). It is highly expressed in the cerebral cortex, cerebellum and brain stem, with low expression in the hippocampus and hypothalamus (Kako et al., 2000).

In addition to the metallochaperone-mediated intracellular copper transport, copper can bind also to glutathione (GSH) for transfer to cystein-rich metallothioneins (MT) for storage (Freedman et al., 1989). Notably, astrocyte secretion of MT3 into the synaptic cleft regulates the availability of copper ions released during neurotransmission (Uchida et al., 2002), and confers neuroprotection by removal of copper from redox-active Aβ:Cu^2+^ complexes abundant in AD brains (Meloni et al., 2008). The importance of GSH in neuronal copper homeostasis is highlighted by the exquisite sensitivity of cultured primary cortical neurons to trace amounts of extracellular copper after GSH depletion. The neurotoxic effect is postulated to involve the generation of Cu^+^ and ensuing free radical mediated oxidative stress (White et al., 1999a; White and Cappai, 2003).

Intracellular copper concentration is further maintained by the export of excess copper via P-type ATPases ATP7A and ATP7B. In the brain, the expression and distribution of ATP7A and ATP7B are developmentally regulated (Barnes et al., 2005; El Meskini et al., 2005; Niciu et al., 2006). In an adult brain, their expression is particularly abundant in neurons from copper-rich brain regions such as the hippocampus, olfactory bulb, cerebellum and choroid plexus (Iwase et al., 1996; Saito et al., 1999; Barnes et al., 2005; Niciu et al., 2006; Choi and Zheng, 2009; Davies et al., 2013). Notably, the co-enrichment of copper and ATP7A in the hippocampus highlights their critical participation in neurotransmission, and potential involvement in learning and memory. Low levels of ATP7A are present also in a sub-population of astrocytes, microglia, myelinating oligodendrocytes, tanycytes, and endothelial cells (Niciu et al., 2006). In the rodent cerebellum, ATP7A and ATP7B are reported to have a distinct cell and developmental specific expression. ATP7B is expressed constitutively in Purkinje neurons, where it functions to deliver copper to CP; whereas there is a developmental shift in ATP7A expression from the Purkinje neurons to Bergmann glia (Barnes et al., 2005). However, in the post-mortem human adult brain, both ATP7A and ATP7B are strongly expressed in Purkinje neurons, but not in Bergmann glia (Davies et al., 2013). At a cellular level, ATP7A, and ATP7B are localized at the TGN, and undergo copper-responsive redistribution to the basolateral and apical cell surface, respectively, where they mediate copper efflux (Niciu et al., 2006; Davies et al., 2013). Additionally, ATP7A localizes to the axon in maturing olfactory receptor neurons, where it has a putative role in axonal outgrowth and synaptogenesis (El Meskini et al., 2005, 2007).

## Brain cholesterol homeostasis

Cholesterol is a fundamental component of the brain. It is an integral structural constituent of cellular membranes of all body cells and a precursor of a number of sterols, hormones, and vitamins. Perturbed brain cholesterol balance can affect membrane dynamics and stability, and contributes to neuronal degeneration, loss of synaptic plasticity and neurotransmission (Koudinov and Koudinova, 2001, 2005). Indeed, neurons may require a threshold level of cholesterol synthesis as statin treatment reduced synapse density and impaired synaptic vesicle release from hippocampal neurons (Mailman et al., 2011). Therefore, it is vital for synaptogenesis, dendritogenesis, axonal guidance, and brain cell signaling events (Mauch et al., 2001; Hering et al., 2003; Goritz et al., 2005; Ko et al., 2005; Guirland and Zheng, 2007; Stottmann et al., 2011). Furthermore, the importance of cholesterol to normal brain development, structure and function is exemplified in neurodegenerative disorders such as Niemann-Pick type C (NP-C) disease, Tangier disease, Smith-Lemi-Opitz Syndrome, Huntington's disease, PD, and AD, that are associated with either genetic or regulatory defects in cholesterol metabolic pathways.

The brain has a disproportionately high cholesterol content to total body mass ratio. The human brain is ~2% of total body mass, yet it contains about a quarter of total body cholesterol. Almost the entire pool of brain cholesterol (>99.5%) is present in the unesterified form, with a distribution rank order of myelin (70%) > glia (20%) > neurons (10%) [reviewed in Björkhem and Meaney (2004) and Dietschy and Turley (2004)]. Brain cholesterol represents a distinct pool from that in the peripheral circulation, which is efficiently segregated by the BBB. Experimental evidence, using ^14^C- or ^2^H-labeled cholesterol, demonstrated that there is negligible exchange of cholesterol between the brain and plasma. In animal models with an intact BBB, uptake of dietary ^2^H-labeled cholesterol into the brain is less than 1% (Meaney et al., 2000). Moreover, in terminally ill human patients with potentially compromised BBB, the detectable influx of ^14^C-labeled cholesterol from peripheral circulation was negligible (Chobanian and Hollander, 1962). Thus, the primary source of brain cholesterol is *de novo* synthesis in both neuronal and glial cells, via the mevalonate pathway, mainly in the endoplasmic reticulum (ER) [reviewed in Buhaescu and Izzedine (2007) and Goldstein and Brown (1990)].

### Brain cholesterol synthesis

The rate of brain cholesterol synthesis is developmentally regulated. The highest rate of cholesterol synthesis occurs during the early postnatal period and early life, a critical time for brain development (Zhang et al., 1996; Dietschy and Turley, 2004). The primary site of cholesterol synthesis is in cholesterol-rich oligodendrocytes that form the insulating myelin sheath around axons. High levels of cholesterol in myelin is required to increase axonal electrical resistance but decrease the capacitance of the surrounding plasma membrane, effectively acting as a barrier to dissipation of electrical impulses across the membrane, whilst promoting transmission down the axon (Snipes and Suter, 1997; Saher et al., 2005). Thus, cholesterol and myelination are essential to neurotransmission that can influence cognitive and motor activities. Therefore, it is no coincidence that the most productive period of myelination parallels that of cholesterol synthesis (Saher et al., 2005). The impact of cholesterol-deficiency and hypomyelination on brain development is evident in neurodegenerative disorders of cholesterol metabolism such as Smith-Lemi-Opitz Syndrome, cerebrotendinous xanthomatosis, hereditary spastic paresis type SPG5, and NP-C, which present clinical symptoms that include mental retardation, dementia, and ataxia [reviewed in Björkhem et al. (2010) and Ikonen (2006)].

The rate of brain cholesterol synthesis declines with brain maturation and age. To preserve a constant level of cholesterol, it has been postulated that in the adult brain, cholesterol is efficiently recycled. Daily sterol turnover in both mouse and human brains is about 20-fold lower compared with that in whole body (Dietschy and Turley, 2004). The estimated half-life of cholesterol in rat brains is about four to six months (Andersson et al., 1990; Björkhem et al., 1997), and in human brains, is estimated to be 5 years (Björkhem et al., 1998). In comparison, in the periphery, the half-life of rat hepatic cholesterol is only about 4 days (Andersson et al., 1990) and ^14^C-labeled cholesterol in human plasma has a reported half-life of about 6 days (Bekersky et al., 2001).

In the adult brain, cholesterol synthesis is not limited to oligodendrocytes, the major center of brain cholesterol production. Attenuated cholesterol synthesis occurs in neurons and astrocytes, but the extent of endogenous cholesterol production in these cells is poorly understood. It has been proposed that in the post-developmental period, it is more energetically efficient for neurons to minimize cholesterol production in favor of electrical impulse propagation that is vital to neurotransmission; and to satisfy neuronal cholesterol requirements, cholesterol is imported from glial cells (Pfrieger, 2003). This hypothesis is supported by experiments involving *in vivo* neuronal conditional inactivation of squalene synthase, the rate-limiting enzyme in the cholesterol synthesis pathways dedicated to sterol formation (Fünfschilling et al., 2007, 2012). Neighboring astrocytes rescued defects in neuronal projection caused by the enforced neuronal cholesterol deficiency. In contrast, the mouse embryonic brain showed a greater demand for cholesterol and it is highly sensitive to cholesterol deficiency. Exogenous cholesterol imported from microglia only partially compensated for the embryonic neuronal cholesterol deficit. Consequently, the mouse pups were born with extensive neuronal degeneration at birth (Fünfschilling et al., 2012). Nevertheless, these observations do not preclude endogenous cholesterol synthesis in neurons. In the adult mouse brain cortical, hippocampal and cholinergic neurons, high expression of key enzymes involved in the first and last step of cholesterol synthesis, 3-hydroxy-3-methylglutaryl-coenzyme A reductase (HMG-CoA reductase) and 7-dehydrocholesterol reductase (DHCR7), respectively, suggest cholesterol synthesis occurs in these neurons (Korade et al., 2007). At present, the requirement for endogenous cholesterol production in these neurons is unclear.

### Brain cholesterol transport: ApoE

The inter- and intra-cellular transfer of cholesterol in the brain involves a multitude of apolipoproteins, lipoprotein receptors and lipid transporters. Apolipoprotein E (ApoE), a 299-amino acid glycoprotein, is the primary apolipoprotein involved in the regulation of lipid transport in both the plasma and the CSF [reviewed in Hauser et al. (2011), Holtzman et al. (2012) and Leduc et al. (2010)]. In humans, there are three major ApoE isoforms: E2, E3, and E4, encoded by polymorphic *APOE* alleles *ε2*, *ε3*, *ε4*, respectively. These isoforms are distinguished by their combination of cysteine and arginine residues at amino acid positions 112 and 158. ApoE3 with the highest allelic frequency (50–90%) is generally considered to be the “parental” form with C112 and R158, whereas in ApoE2, both of these amino acid residues are cysteines, and in ApoE4, both are arginines (Utermann et al., 1984; Mahley, 1988). In contrast, only an ApoE3-equivalent form of ApoE is present in all other species (Hatters et al., 2006). The *APOE-ε4* genotype is the strongest known genetic risk factor for sporadic late-onset AD (Mahley, 1988; Bertram and Tanzi, 2012). Hence, ApoE is the best characterized of all known apolipoproteins, which include ApoA-I, ApoD, and ApoJ (clusterin) (Borghini et al., 1995). Of these other apolipoproteins, ApoJ has gained increasing research interest in recent years with its emergence as another key genetic risk factor for AD from genome-wide association studies (Bertram and Tanzi, 2009; Harold et al., 2009).

Similar to cholesterol, ApoE in the brain derives primarily from *de novo* synthesis in glial cells, mostly in astrocytes (Boyles et al., 1985; Pitas et al., 1987a), and there is effectively no exchange with ApoE from the plasma due to lack of permeability through the BBB under normal conditions (Linton et al., 1991). Paradoxically, the stability of the BBB requires ApoE, which exerts an isoform-dependent influence on the integrity of cerebrovasculature (Methia et al., 2001; Nishitsuji et al., 2011; Bell et al., 2012). Minor amounts of ApoE are produced in neurons and microglia under normal conditions, but selective up-regulation of neuronal ApoE can occur under pathological conditions such as stroke and AD (Xu et al., 1999; Aoki et al., 2003). The functional importance of neuronal ApoE under such conditions is not well understood, but indicates a potential role for neuron-derived ApoE to enhance local cholesterol recycling to facilitate neuronal repair.

Astrocyte-derived ApoE has a dual role in cholesterol transport. It is instrumental to the delivery of cholesterol to distal axons for synaptogenesis, dendritogenesis, axonal guidance, signaling, and release of synaptic vesicles in neurotransmission (de Chaves et al., 1997; Posse De Chaves et al., 2000; Mauch et al., 2001; Hering et al., 2003; Goritz et al., 2005). The concomitant neuronal uptake of ApoE and cholesterol present in spherical and discoidal high-density lipoprotein (HDL)-like particles is via a family of low-density lipoprotein receptors, which include low-density lipoprotein receptor (LDLR), LDLR-related protein 1 (LRP1), and ApoE receptor 2 (ApoER2). The structural conformation of ApoE, as dictated by its isoform and lipidation status, determines its binding affinity to LDL receptors (Hatters et al., 2006). Experimental evidence revealed that a positively charged amino acid at position 158 in ApoE is essential for effective binding to LDL receptors (Innerarity et al., 1984; Dong et al., 1996). It is noteworthy that ApoE2 is defective in binding to LDLR, which is due to reduced positive electrostatic potential with a cysteine at this position (Weisgraber et al., 1982; Lund-Katz et al., 2001), yet it is the most protective ApoE isoform against AD. ApoE3 and ApoE4 bind to LDLR with similar affinity. Furthermore, lipidation of ApoE is essential for binding to LDL receptors, and an *in vitro* lipid binding experiment demonstrated that ApoE4 has the highest lipid binding affinity, although all isoforms have a similar lipid binding capacity (Saito et al., 2003).

In addition to its role in neuronal cholesterol delivery, ApoE is essential also to cholesterol efflux from neurons and astrocytes. In this pathway, ApoE functions as an acceptor of cellular cholesterol and other lipids from cell surface ATP-binding cassette transporters such as ABCA1 and ABCG1 (Kim et al., 2007, 2008; Minagawa et al., 2009). This process of ApoE lipidation then increases ApoE affinity for LDL receptors for delivery of cholesterol to neurons. The effectiveness of ApoE as a cholesterol acceptor is isoform-specific. *In vitro* experimental evidence showed that highest cholesterol efflux activity is achieved in both neuronal and astrocyte cultures in the presence of exogenous ApoE2 (Michikawa et al., 2000). Therefore, the neuroprotective capacity of ApoE2 may be partially explained by its proficiency in facilitating cellular cholesterol efflux, despite being ineffective in cholesterol delivery due to inefficient binding to LDLR.

### Intraneuronal cholesterol transport

The intracellular fate of ApoE-bound cholesterol in neurons is believed to follow that of the classical clathrin-dependent receptor-mediated endocytic pathway [reviewed in Goldstein and Brown (2009)]. HDL-like particles containing cholesterol-bound ApoE/LDLR complex associate with the clathrin-coated vesicles, which initially fuse with the sorting endosomes, where the complex dissociates and LDLR is then either recycled back to the cell surface or delivered to lysosomes for degradation. HDL-like particles are further transported to the late-endosomes/lysosomes, where the acidic environment of these compartments promotes the dissociation of the HDL-like particles, liberating ApoE and cholesterol. The subsequent mobilization of cholesterol from late-endosomes/lysosomes involves NPC1 and NPC2 proteins (Vanier, 2010). Fibroblast cells extracted from NP-C patients with a deficiency in either NPC1 and/or NPC2 proteins show an entrapment of unesterified cholesterol in late-endosomes/lysosomes (Pentchev et al., 1985; Kruth et al., 1986). This abnormal cholesterol accumulating phenotype is present also in both NP-C human and mouse model brains (Karten et al., 2002; Distl et al., 2003; Treiber-Held et al., 2003). More recently, *in vitro* experiments that demonstrate direct binding and interactions between NPC1, NPC2, and cholesterol provided further support for a critical role for NPC1 and NPC2 in intracellular cholesterol transport (Infante et al., 2008a,b,c; Kwon et al., 2009; Wang et al., 2010). It has been postulated that NPC1 and NPC2 mediated cholesterol egress from late-endosomes/lysosomes can be diverted to either the ER for esterification by acetyl-coenzyme A:cholesterol acetyltransferase (ACAT) for storage of the excess in cytosolic lipid droplets, or to the plasma membrane for export by cholesterol transporters (*e.g*., ABCA1 and ABCG1). Genetic defects in ABCA1 causes Tangier disease (Kolovou et al., 2006), which exhibits a cholesterol accumulating phenotype similar to that found in NP-C and is characterized by HDL-deficiency. There are limited reports on the effects of ABCA1 mutations in the central nervous system (Pietrini et al., 1990; Negi et al., 2013), with premature atherosclerosis and neuropathy being the best documented symptoms of Tangier disease.

### Brain cholesterol export

Cholesterol cannot be degraded. At present, there are two known pathways for brain cholesterol elimination. One route of brain cholesterol removal is via ApoE secretion into the CSF and export from the brain at a rate of 1–2 mg/day by an unknown mechanism (Pitas et al., 1987b; Xie et al., 2003). Cholesterol removed by this pathway is believed to originate primarily from glial cells. Alternatively, the main route of brain cholesterol elimination, primarily of neuronal origin, is via conversion of cholesterol to BBB-permeable oxidized cholesterol species (oxysterols). The primary brain oxysterol species is 24S-hydroxycholesterol (24OHC), the product of 24S-hydroxylase (CYP46) catalyzed addition of a hydroxyl group to the 24-position of cholesterol (Lutjohann et al., 1996; Björkhem et al., 1997, 1998; Lund et al., 1999, 2003; Lutjohann and von Bergmann, 2003). The rate of 24OHC movement from the brain into the circulation is estimated at 6–7 mg/day (Lutjohann et al., 1996; Björkhem et al., 1998). A corresponding rate of uptake in the liver has led to the hypothesis that 24OHC is an exclusive product of the brain, and its concentration in the circulation is reflective of brain cholesterol metabolism (Bretillon et al., 2000; Lutjohann and von Bergmann, 2003). In the liver, 24OHC is further converted to bile acids for excretion from the body (Björkhem et al., 2001). By analogy with 24OHC penetration of the BBB, 27-hydroxycholesterol (27OHC) produced by peripheral cells can enter the brain from circulation at a rate of 5 mg/day (Heverin et al., 2005). There are no known oxysterol transporters, and the transfer of oxysterols across cell membranes is hypothesized to be driven by the concentration gradient (Björkhem et al., 2009).

Oxysterols play an important part in regulation of whole body cholesterol homeostasis. They can activate liver X receptors (LXRs), LXRα, and LXRβ [reviewed in Björkhem (2013)], which regulate a number of genes in the cholesterol metabolic pathway including ABCA1, ABCG1, SREBP-1, and ApoE. Interestingly, cultured glial cells respond to a greater extent to LXR agonist stimulation of LXR target gene expression and cholesterol efflux compared with cultured neuronal cells (Whitney et al., 2002). Thus, LXR and 24OHC are connected in a feedback mechanism to maintain a net cholesterol export, where an increased export of neuronal cholesterol in the form of 24OHC activates cholesterol export from glial cells for delivery to neurons (Pfrieger, 2003). In addition to LXRs, other members of the same nuclear receptor family that also regulate genes in the cholesterol metabolic pathway include peroxisome proliferator-activated receptor γ (PPARγ) and retinoid X receptors (RXRs).

## Crosstalk between copper and cholesterol metabolism

The interaction between copper and cholesterol metabolic pathways in the brain is an area of limited research. The majority of current investigations center mainly on peripheral changes. Dietary manipulations in animal studies revealed an inverse relationship between copper and cholesterol in their respective peripheral concentrations. Rats and rabbits fed a high cholesterol diet have decreased hepatic copper concentration compared with animals on a control diet (Klevay, 1988; de Wolf et al., 2003a,b). Increased bilirubin secretion has been proposed as the potential mechanism underlying cholesterol induced hepatic copper reduction (de Wolf et al., 2003b). However, in the Watanabe heritable hyperlipidemic rabbits, an animal model for familial hypercholesterolemia, there is no inverse correlation with copper, which is significantly elevated (Allain et al., 1989). These observations indicate that dietary and genetic changes in cholesterol have differential effects on copper metabolism. It is conceivable that dietary cholesterol alters cholesterol homeostasis in a manner that affects cellular membrane fluidity and dynamic, which impacts on the structure and function of membrane-localized copper transporters. For example, pharmacological depletion of cellular cholesterol levels using the cholesterol sequestering agent methyl-β-cyclodextrin (MβCD) resulted in a shift of the copper transporter CTR1 in HeLa cells from a perinuclear localization to a more diffused vesicular and plasma membrane distribution (Klomp et al., 2002), and thus cause a change in cellular copper balance. However, the exact mechanism underlying the impact of altered cholesterol metabolism on copper homeostasis remains to be elucidated.

**Figure 1:**
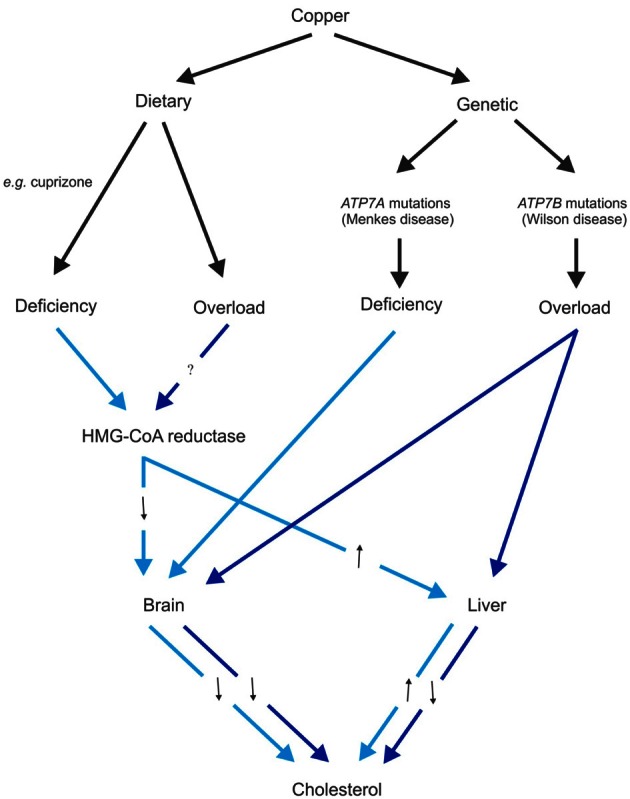
**Schematic diagram of the effects of dietary and genetic copper manipulation on cholesterol levels, based on available data from animal models.** Light blue and dark blue arrows represent pathways associated with copper deficiency and overload conditions, respectively. Black arrows denote either increased (↑) or decreased (↓) 3-hydroxy-3-methylglutaryl-coenzyme A (HMG-CoA) reductase expression or cholesterol levels as a consequence of altered copper levels.

In animals fed a copper deficient diet, peripheral cholesterol synthesis is stimulated via up-regulation of HMG-CoA reductase and SREBP-1, and they develop hypercholesterolemia (Carr and Lei, 1989; Yount et al., 1990; Lei, 1991; al-Othman et al., 1994; Tang et al., 2000). In contrast, brain cholesterol synthesis and myelination is sensitive to dietary and genetic copper deficiencies (Figure 1). Mice given dietary supplement of cuprizone, a copper chelator, showed a significant down-regulation of HMG-CoA reductase mRNA expression and cholesterol synthesis, and consequently, severe demyelination, affecting particularly the corpus callosum (Jurevics et al., 2001, 2002). These effects are reversible by removal of cuprizone from the diet. Similarly, profound brain copper deficiency in Menkes disease, due to absent or defective activity of the ATP7A copper transporter, is associated also with significantly reduced brain cholesterol and extensive myelin defects (Hara and Taketomi, 1986). Conversely, copper overload in the *Atp7b*^−/−^ mouse model of Wilson disease is associated also with reduced brain and hepatic concentrations of cholesterol, lathosterol, desmosterol, 8-hydrocholesterol, and 7-dehydrocholesterol (Sauer et al., 2011). These changes are consistent with down-regulation of cholesterogenic gene expression and reduction in nuclear receptor transcription factors farnesoid X receptor (FXR) and glucocorticoid receptor (GR) in the liver of the same animal model (Huster et al., 2007; Wilmarth et al., 2012). However, the decrease in serum cholesterol observed in animal models of Wilson disease is not recapitulated in a study of human Wilson disease patients (Seessle et al., 2011). Also inconsistent with *in vivo* animal studies, a microarray analysis of *in vitro* human macrophage culture treated with copper found up-regulation of seven cholesterolgenic genes, which include HMG-CoA synthase, squalene synthase, and LDLR (Svensson et al., 2003). These observations suggest that there may be tissue- and species-specific differences in cholesterol metabolism in response to copper. There is clearly a close and complex relationship between the copper and cholesterol metabolic pathways. The full impact of altered copper homeostasis with regard to cholesterol metabolism remains to be fully explored.

## Disintegration of copper and cholesterol metabolism in Alzheimer's disease

Late-onset AD is a complex multifactorial disorder, and to date, the exact etiology remains elusive. There are largely independent bodies of literature documenting the disruption to copper and cholesterol homeostasis and how they may contribute to the development and progression of AD. Observations from epidemiological studies of changes in plasma copper and cholesterol as discriminating markers for AD have been inconsistent (Jarvik et al., 1995; Notkola et al., 1998; Gonzalez et al., 1999; Squitti et al., 2002, 2003, 2005, 2006, 2007; Reitz et al., 2004, 2008; Mielke et al., 2005; Pajonk et al., 2005; Kessler et al., 2006; Kivipelto and Solomon, 2006; Solomon et al., 2007; Zambon et al., 2010; Rembach et al., 2013). The inherent genetic and environmental heterogeneity in different human populations may contribute to the ambiguous observations. Nevertheless, epidemiological and animal studies found that a high fat diet is associated with cognitive decline and increased risk for AD (Morris et al., 2003; Sparks, 2008). High copper consumption with a high saturated and *trans* fat diet further accelerates the cognitive decline (Morris et al., 2006). The latter study suggests that the association is independent of dietary cholesterol, zinc and iron; but this study did not account for potential effects due to stimulation of endogenous cholesterol synthesis. An AD cholesterol-lowering treatment trial involving atorvastatin reported an increase in plasma CP as a measure of copper (Sparks et al., 2005), further suggesting an interaction between copper and cholesterol homeostasis in AD. Collectively, these studies, which mostly document peripheral copper and cholesterol changes, indicate a synergistic failure of copper and cholesterol metabolic pathways that contributes to cognitive decline and pathogenesis of AD.

### Interactions of copper and cholesterol with APP and Aβ

Amyloid plaques that are characteristic of AD brains contain high concentrations of the Aβ peptide, cholesterol and transition metals (copper, iron and zinc) (Glenner and Wong, 1984; Masters et al., 1985; Lovell et al., 1998; Dong et al., 2003; Miller et al., 2006; Panchal et al., 2010). Central to the formation of amyloid plaques is the processing of APP, a glycosylated type I transmembrane protein. There are two alternative APP processing pathways, commonly referred to as the amyloidogenic or non-amyloidogenic pathway (Thinakaran and Koo, 2008). The release of Aβ by sequential β- and γ-secretase cleavage of APP in the amyloidogenic pathway is most relevant to AD pathogenesis. Conversely, non-amyloidogenic processing of APP involves sequential α- and γ-secretase cleavages to release a non-neurotoxic p3 peptide. Cleavage by γ-secretase in both pathways liberates a common C-terminal fragment known as APP intracellular domain (AICD), which can translocate to the nucleus and function as a transcription factor.

**Figure 2:**
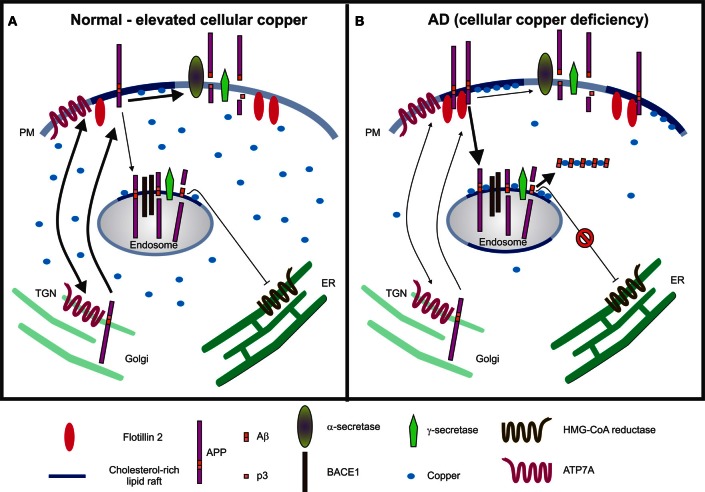
**Schematic models illustrating the interactions between copper, cholesterol and APP metabolism. (A)** Under normal and/or elevated cellular copper conditions, there is an increased translocation of APP and ATP7A from the *trans*-Golgi network (TGN) to the plasma membrane (PM), where they participate in copper efflux. The majority of the APP molecules undergo non-amyloidogenic processing via α-secretase cleavage at the cell surface in non-lipid raft membrane domains, which produces a non-toxic p3 fragment. It is expected that a basal level of β-cleavage of APP occurs in cholesterol-rich lipid raft domains of acidic endosomes enriched in BACE1, the β-secretase. Aβ_40_, the product of the sequential cleavage of APP by β- and γ-secretases, is proposed to inhibit 3-hydroxy-3-methylglutaryl-coenzyme A (HMG-CoA) reductase activity as a part of a feedback mechanism by which cholesterol-dependent regulation of APP occurs (Grimm et al., 2005). **(B)** In contrast, under conditions of copper deficiency, such as that present in Alzheimer's disease (AD), there may be increased APP interactions with flotillin-2 at the PM, increased APP partitioning into lipid rafts, enhanced internalization of APP to BACE1-rich endosomes for amyloidogenic processing and generation of Aβ in lipid rafts. The combination of Aβ, cholesterol and copper in lipid rafts fosters a favorable environment for the formation of redox-active Aβ:Cu^2+^ complexes, which can catalytically oxidize cholesterol, and may potentiate neurotoxicity by generating H_2_O_2_ and toxic oxysterol species. Although there may be an increased production of Aβ, the formation of Aβ:Cu^2+^ oligomers may result in reduced availability of Aβ to inhibit HMG-CoA reductase activity and consequently, increased cholesterol synthesis.

Cholesterol is a widely recognized key regulator of APP processing. Amyloidogenic processing of APP preferentially occurs in cholesterol- and glycosphingolipid (GSL)-rich lipid raft microdomains (Simons and Ikonen, 1997; Ehehalt et al., 2003; Kawarabayashi et al., 2004; Vetrivel et al., 2004; Hooper, 2005; Cordy et al., 2006; Cheng et al., 2007; Schneider et al., 2008; Thinakaran and Koo, 2008; Hung et al., 2009; Vetrivel and Thinakaran, 2010). APP interactions at the cell surface with flotillin-2, a lipid raft protein (Schneider et al., 2008), and cholesterol via its C-terminal domain (Beel et al., 2008) are critical to its partitioning into lipid rafts and trafficking (Figure 2). Internalization of APP from the cell surface to BACE1 (β-secretase)-rich acidic endosomes is cholesterol-sensitive and pro-amyloidogenic (Refolo et al., 1995; Vassar et al., 1999; Schneider et al., 2008; Cossec et al., 2010; Marquer et al., 2011). Alternatively, cholesterol depletion triggers destabilization of lipid rafts, decreased APP internalization and lipid raft localization, inhibition of β- and γ-secretase activities, and reduced Aβ production (Simons et al., 1998; Fassbender et al., 2001; Riddell et al., 2001; Ehehalt et al., 2003; Vetrivel et al., 2004, 2005; Urano et al., 2005; Cordy et al., 2006; Schneider et al., 2008; Won et al., 2008; Cossec et al., 2010; Marquer et al., 2011). Two common agents used to deplete cholesterol are methyl-β-cyclodextrin (MβCD) and statins. MβCD extracts cholesterol from cellular membranes. Statins are inhibitors of HMG-CoA reductase, thereby achieving cholesterol reduction by interfering with endogenous cholesterol synthesis via the mevalonate pathway. Given the pro-amyloidogenic nature of cholesterol, it has been considered as a potential therapeutic target for treatment of AD. A number of clinical trials have investigated the beneficial effects of statins [reviewed in Shepardson et al. (2011a,b)]. The outcomes are variable due to the pleiotropic nature of statins, which differ in their BBB permeability and additional off-target effects on the isoprenylation pathway.

Copper also modulates APP processing (Borchardt et al., 1999; Cater et al., 2008; Hung et al., 2009). Conditions of copper deficiency, such as that found in brain tissues from AD patients and transgenic mouse models promote amyloidogenic processing of APP (Deibel et al., 1996; Maynard et al., 2002, 2006; Bayer et al., 2003; Phinney et al., 2003; Magaki et al., 2007; Cater et al., 2008) in cholesterol-rich lipid rafts (Hung et al., 2009) (Figure 2). Conversely, increasing cellular copper levels via dietary, pharmacological or genetic manipulations favors the non-amyloidogenic processing of APP and inhibits Aβ production (Borchardt et al., 1999; Cherny et al., 2001; Bayer et al., 2003; Phinney et al., 2003; White et al., 2006; Adlard et al., 2008; Cater et al., 2008; Donnelly et al., 2008; Hung et al., 2009). Analogous to cholesterol depletion, increased cellular copper reduces the association of flotillin-2 with lipid rafts (Hung et al., 2009), retards APP internalization and attenuates Aβ production (Hung et al., 2009; Acevedo et al., 2011). It can be envisaged that the copper-dependent effect on APP processing may be achieved via regulation of cholesterol synthesis (see Section Crosstalk between copper and cholesterol metabolism). The alteration in copper-induced APP redistribution and processing may stimulate a compensatory response in the up-regulation of APP mRNA and protein expression (Armendariz et al., 2004; Bellingham et al., 2004b; Cater et al., 2008).

In a reciprocal manner, APP participates in the regulation of copper and cholesterol metabolism. A dual mechanism is involved in APP regulation of cholesterol metabolism. The binding of cholesterol to APP C-terminal domain promotes its amyloidogenic processing, and the release of Aβ and AICD. Experimental evidence demonstrates that Aβ_40_ inhibits the activity of HMG-CoA reductase, the rate limiting enzyme in the cholesterol synthesis pathway, so that APP deficiency results in cholesterol accumulation (Grimm et al., 2005). An additional mechanism of cellular cholesterol reduction is achieved by translocation of AICD into the nucleus, where it down-regulates LRP1 expression by transcription suppression (Liu et al., 2007). Thus, APP is proposed as a cholesterol sensor to control cellular cholesterol levels and is own processing (Beel et al., 2008). Additionally, APP is proposed to regulate copper efflux. This hypothesis evolved from evidence that APP expression inversely correlates with copper levels in cell and animal models (White et al., 1999b,c; Maynard et al., 2002; Phinney et al., 2003; Bellingham et al., 2004a). Over-expression of APP or its C-terminal domain (C100) that contains the Aβ sequence is associated with copper deficiency in transgenic mouse models of AD (Maynard et al., 2002; Bayer et al., 2003; Phinney et al., 2003). Conversely, APP deficiency causes copper accumulation (White et al., 1999c; Bellingham et al., 2004a). The concomitant rise in copper and cholesterol in APP knockout animals suggests an interaction between these pathways that becomes uncoupled in its absence.

Oxidative stress is a pathological feature of AD. It is noteworthy that the inverse nature of copper distribution to cholesterol-rich lipid rafts relative to cellular copper concentrations fosters an environment conducive to oxidative stress (Hung et al., 2009) (Figure 2). Lipid raft co-enrichment of Aβ and copper favors the formation of redox-active Aβ:Cu^2+^ complexes that catalytically generate H_2_O_2_ via reduction of Cu^2+^ to Cu^+^ (Atwood et al., 1998, 2004; Huang et al., 1999; Cuajungco et al., 2000; Hung et al., 2009). Cholesterol and long-chain fatty acids in lipid rafts are obvious electron donors that participate in this reaction (Opazo et al., 2002; Haeffner et al., 2005; Murray et al., 2005, 2007), and consequently are converted to oxysterols and lipid peroxidation products, such as 7β-hydroxycholesterol and 4-hydroxynonenal (HNE), which are present in abundance in the brains of human AD cases and mouse models (Opazo et al., 2002; Murray et al., 2005, 2007; Nelson and Alkon, 2005; Puglielli et al., 2005; Yoshimoto et al., 2005; Jiang et al., 2007). A neurotoxic vicious cycle then ensues, where oxidatively modified lipids stimulate the formation of Aβ oligomers complexed with copper and enter a continual pathological redox cycle oxidizing cholesterol and other membrane lipids (Koppaka and Axelsen, 2000; Barnham et al., 2003; Ciccotosto et al., 2004; Murray et al., 2005, 2007; Tickler et al., 2005; Liu et al., 2008). Furthermore, *in vitro*, oligomers of Aβ:Cu^2+^ can penetrate artificial membrane systems to form cation channels (Bhatia et al., 2000; Curtain et al., 2001, 2003; Lin et al., 2001). Collectively, these observations suggest that a combination of copper, soluble Aβ oligomers and cholesterol may potentiate neurotoxicity via lipid oxidation and altered membrane integrity.

Sporadic AD is often simulated by cholesterol feeding of animal models (rabbits, beagles, and mice) to induce AD-like pathologies and cognitive deficits (Ghribi et al., 2006; Woodruff-Pak et al., 2007; Ghribi, 2008; Sparks, 2008; Lu et al., 2009, 2006; Sparks et al., 2011). In rabbit brains, amyloid plaque-like structures are detected in the hippocampus and temporal lobe, similar to that in human AD brains (Sparks and Schreurs, 2003; Sparks, 2004; Sparks et al., 2006; Woodruff-Pak et al., 2007). Additional consumption of trace amounts of copper (0.12 ppm) in drinking water, but not zinc or aluminum, accelerated the cognitive decline with exacerbated amyloid pathology in cholesterol-fed rabbits suggestive of an interaction between dietary cholesterol and copper in this event (Sparks et al., 2006). In contrast, a normal diet with copper only treatment had no significant effect on amyloid pathology and cognition (Sparks and Schreurs, 2003; Lu et al., 2006). However, more recent studies showed conflicting data that copper treatment alone at a higher concentration of 0.24 ppm resulted in increased Aβ-positive neurons in the rabbit model (Deci et al., 2012); and chronic treatment of rodent models at a high dosage (250 ppm) also enhanced the amyloid pathology (Kitazawa et al., 2009; Mao et al., 2012). The impact of higher copper intake on cholesterol homeostasis in these studies is unclear. Potential mechanisms of dietary cholesterol in causing brain Aβ accumulation include increased circulating and brain concentrations of 27-OHC, and up-regulation of APP and BACE1 expression (Ghribi, 2008; Prasanthi et al., 2009; Shafaati et al., 2011; Popp et al., 2012). Copper may contribute to augmentation of dietary cholesterol-induced amyloid pathology and neurotoxicity by further up-regulating APP and BACE1 expression, increasing oxidative stress and activation of inflammatory pathways (Lu et al., 2006, 2009; Lin et al., 2008). An alternative hypothesis for copper-induced aggravation of amyloid pathology in cholesterol-fed animals is by impeding the clearance of Aβ complexed to ApoE-cholesterol from the brain for removal from the body by the liver (Sparks, 2007). However, the exact biochemical mechanism remains to be elucidated.

### Link between copper and ApoE

There is an emerging literature linking the main extracellular cholesterol transporter ApoE and copper, alluding to a potential protective role for ApoE against copper-mediated toxicity and/or copper regulation. The antioxidant activity of ApoE is isoform-specific, in the order of E2>E3>E4, with ApoE2 demonstrating a >2-fold higher activity relative to ApoE4 (Miyata and Smith, 1996). The same study showed that ApoE binds metals including copper, zinc and iron, with highest affinity for copper, and it inhibits copper-mediated lipoprotein oxidation. The authors proposed that copper sequestration may account for the antioxidant activity of ApoE. Moreover, copper-induced Aβ accumulation and aggregation is most pronounced in the presence of ApoE4 compared with ApoE2 and ApoE3 (Moir et al., 1999). Hence, the ability of ApoE to bind to copper with high affinity is a potential mechanism for mitigating copper-mediated lipoprotein oxidation and copper-induced Aβ aggregation (Miyata and Smith, 1996; Moir et al., 1999). The precise ApoE copper-binding mechanism is unknown. Potential sites that may coordinate copper include the methionine residues within the N-terminal four-helix bundle (Miyata and Smith, 1996). The cysteines of ApoE2 at amino acid positions 112 and 158 have the potential to bind copper, while ApoE4, which lacks the cysteine residues, is proposed to have a reduced copper binding capacity and thus, decreased ability to clear Aβ from the brain (Brewer, 2009). However, this may not be feasible based on apoE structural studies [reviewed in Hatters et al. (2006)], which suggest that these cysteine residues are positioned in an opposing orientation.

An ApoE-copper connection was further supported by the finding that in Wilson disease patients with the common H1069Q mutation in the copper transporter ATP7B, an earlier onset of symptoms is associated with the *APOE-ε4* genotype (Litwin et al., 2012). In comparison, *APOE-ε3* genotype is associated with a significant delay in the onset of Wilson disease symptoms (Schiefermeier et al., 2000; Wang et al., 2003). The authors proposed that ApoE4 is less effective than ApoE2/ApoE3 as an antioxidant so that ApoE4 patients are more susceptible to copper toxicity. These studies further suggest a role for ApoE in copper regulation and in influencing Wilson disease phenotypes, but the exact mechanism remains unclear.

It is noteworthy that ApoJ also interacts with the ATP7A and ATP7B copper transporters that are defective in Menkes and Wilson diseases, respectively (Materia et al., 2011, 2012). ApoJ facilitates their degradation and thus can influence cellular copper levels. However, the integration of these apolipoproteins' lipid transport and Aβ clearance activities with their role in copper regulation remains to be further clarified.

### Copper, cholesterol, and tau

NFTs, the other distinguishing pathological hallmark of AD, are mainly formed from aggregates of hyperphosphorylated tau protein [reviewed in Avila et al. (2004), Morris et al. (2011), Spires-Jones et al. (2009)]. Tau is best characterized for its role in polymerization and stabilization of neuronal microtubules, and regulation of axonal transport of synaptic vesicles in neurotransmission (Thies and Mandelkow, 2007; Dixit et al., 2008). However, it is increasingly recognized as a multi-functional protein. Recent findings report the release of tau with synaptic vesicles as a part of neurotransmission (Pooler et al., 2013) and it has been ascribed a regulatory role in neuronal signaling pathways (Ittner et al., 2010, 2011). Phosphorylation of tau is well-established as a modification that regulates its activity, particularly with respect to its interactions with microtubules. An imbalance of tau interacting kinase and phosphatase activities is believed to contribute to the genesis of hyperphosphorylated tau abundant in NFTs.

Tau has a peripheral membrane association via its N-terminal projection domain, implicating a role for tau in neurite outgrowth (Brandt et al., 1995). However, there is limited information regarding a direct interaction between tau and cholesterol or other lipids. Lipid raft studies have revealed an AD- and age-dependent enrichment of tau phosphorylated at NFT-associated epitopes in brain tissues from human AD cases and the Tg2576 mouse model (Kawarabayashi et al., 2004). Indeed, there is a co-localization of cholesterol and NFTs in the same neuronal populations in AD and NP-C brains (Distl et al., 2001). Pharmacological cholesterol depletion by statin-mediated inhibition of cholesterol synthesis resulted in enhanced tau phosphorylation at NFT-associated epitopes (Fan et al., 2001). These data are suggestive of a role for cholesterol in tau phosphorylation and development of NFTs, although the mechanism involved remains to be elucidated.

Literature on the interaction between copper and tau is also limited. The promoter of the tau (*MAPT*) gene has the potential to be regulated by Sp1, a copper-responsive transcription factor (Heicklen-Klein and Ginzburg, 2000; Song et al., 2008; Bellingham et al., 2009). Over-expression of tau inhibits trafficking of APP to the cells surface (Stamer et al., 2002); and ATP7A traffics to the cell surface along the microtubule network (Cobbold et al., 2004). Therefore, it is plausible that copper-regulated tau expression may be a regulatory mechanism to control copper-responsive trafficking of APP and ATP7A (Stamer et al., 2002; Cobbold et al., 2004; Hung et al., 2009; Acevedo et al., 2011). The functional implications of this regulatory control include NMDA receptor-mediated excitotoxicity, ATP7A-mediated synaptic release of copper and APP metabolism.

Copper is associated with NFTs (Sayre et al., 2000). *In vitro* experiments indicate that adventitious binding of copper to tau is redox-competent and contributes to neuronal oxidative stress through the generation of hydrogen peroxide. Detailed structural and biophysical characterization of the longest human tau isoform established that tau binds to one Cu^2+^ ion per monomer under mild acidic conditions with high affinity (*K*_*d*_ = 0.5 μM) (Soragni et al., 2008). Copper coordination involves the cysteine residues 291 and 322 within the microtubule-binding domain. It is notable that evidence from *in vitro* experiments suggests that copper-mediated tau aggregation is phosphorylation independent; but the manner in which copper may catalyze tau aggregation *in vivo* is unknown.

The effects of copper on tau pathology *in vivo* in AD models also reveal conflicting results. Copper modulation by treating a double transgenic AD mouse model with Cu^II^(gtsm), a bis(thiosemicarbazone) or PBT2, a copper/zinc ionophore, improved tau phosphorylation, Aβ oligomerization and cognition (Adlard et al., 2008; Crouch et al., 2009). However, chronic administration of CuSO_4_ to a triple transgenic AD mouse model caused an increase in cdk5/p25 phosphorylated tau species (Kitazawa et al., 2009). These apparent discrepancies remain to be resolved.

In general, studies that investigate the interaction between copper, cholesterol and tau are lacking. In cholesterol-fed rabbits, copper appears to ameliorate tau pathology in the hippocampus and frontal cortex of cholesterol-fed rabbits, but induces a concomitant rise in plasma tau concentration (Sparks et al., 2011). The mechanism that underlies this observation awaits further studies. Interestingly, in the cholesterol-fed rabbits, the apparent normalization effect of copper on brain tau pathology opposes the negative impact of copper on the amyloid pathology. There is no reasonable explanation for this apparent contradiction at present.

## Conclusions

AD is a complex and multifactorial neurodegenerative disorder, and failures in many biochemical pathways are implicated in the pathogenesis of sporadic late-onset AD. Disruption of copper and cholesterol homeostasis are two major pathological features of AD, but whether abnormalities in copper and cholesterol are a cause or consequence of the disease process is not clear. The finding of impaired copper and cholesterol metabolism in other neurodegenerative diseases such as PD and NP-C highlights the possibility that there are fundamental mechanisms linking the biochemistry of these essential cellular constituents. This review has highlighted the essential requirements for copper and cholesterol for normal brain functions, and has summarized the growing body of evidence documenting the close and complex relationship between copper and cholesterol metabolism. Clearly disturbance to one is associated with dysregulation of the other. The complexity is further enhanced by the interactions of copper and cholesterol with key proteins that are dysregulated or malfunction in AD such as, APP, Aβ and tau; and the heterogeneity of genetic and environmental influences between individuals and populations. For example, there is an intimate interaction between copper and cholesterol in APP metabolism that may explain some of the neurodegeneration evident in AD. Key issues that could be addressed to provide further insight are: (1) the relationship between copper, cholesterol and tau; (2) whether the major risk associated factors ApoE and ApoJ are associated with copper *in vivo* and whether copper is necessary for their function; (3) whether copper is associated with HDL-like particles containing cholesterol-bound ApoE and if so, the functional significance of this in both copper and cholesterol transport and AD. Further research is required for an integrated understanding of the molecular mechanisms connecting copper and cholesterol homeostasis with various players in AD, and how their disintegration may contribute to the development and progression of AD. Such knowledge is critical to the design of effective disease-modifying therapeutic targets.

### Conflict of interest statement

The authors declare that the research was conducted in the absence of any commercial or financial relationships that could be construed as a potential conflict of interest.
